# Nucleated Erythrocytes: Linking Structure, Mechanics, Function, and Stress Responses

**DOI:** 10.3390/ijms27219527

**Published:** 2026-10-25

**Authors:** Tianzhu Yu, Zuobin Wang

**Affiliations:** 1 International Research Centre for Nano Handling and Manufacturing of China, Changchun University of Science and Technology, Changchun 130022, China; 2 Centre for Opto/Bio-Nano Measurement and Manufacturing, Zhongshan Institute of Changchun University of Science and Technology, Zhongshan 528437, China; 3 Ministry of Education Key Laboratory for Cross-Scale Micro and Nano Manufacturing, Changchun University of Science and Technology, Changchun 130022, China; 4 Joint Research Centre for Computer-Controlled Nanomanufacturing, University of Bedfordshire, Luton LU1 3JU, UK

**Keywords:** nucleated red blood cells, human red blood cells, cellular mechanics, oxidative stress, immune response

## Abstract

Red blood cells have traditionally been studied through mature mammalian erythrocytes, which provide a model of highly deformable enucleated cells. However, nucleated erythrocytes retained by birds, fish, amphibians, and reptiles represent a distinct circulating cell strategy that integrates membrane–skeleton organization, cytoplasmic properties, nuclear structure, organelle function, and stress responses. This review uses human mature red blood cells as a mechanical reference rather than a replacement model to examine how conserved and nucleus/organelle-associated mechanisms link erythrocyte structure, mechanics, and function. Current evidence indicates that nucleated erythrocytes can be considered composite cellular systems in which the nucleus may contribute to local mechanical heterogeneity and deformation behavior, although direct causal links to whole-cell mechanical phenotypes remain insufficiently established. Comparative studies across vertebrate models reveal complementary insights into nuclear regulation, metabolic adaptation, immune responses, and stress tolerance. The major challenge is to establish causal relationships between structural remodeling, mechanical changes, and physiological outcomes using integrated mechanical, imaging, and molecular approaches. This review highlights nucleated erythrocytes as valuable models for understanding the relationships among cellular architecture, mechanics, and function beyond the conventional enucleated red blood cell paradigm.

## 1. Introduction

Red blood cells are highly specialized circulating cells whose structure and mechanics are closely linked to their physiological functions [1,2]. Mature red blood cells adopt a biconcave disk structure with a high surface area-to-volume ratio through the removal of the nucleus and most organelles [3]. Meanwhile, the spectrin–actin membrane skeleton maintains their stable morphology and confers extremely high deformability [4], enabling them to repeatedly traverse narrow capillaries and the splenic microenvironment [5]. The review by Kumar et al. further points out that although mature mammalian erythrocytes lack nuclei and most organelles, they still retain complex membrane organization, metabolic regulation and signaling functions [1]. Therefore, when taking human red blood cells as a reference, their comparative advantages can be mainly limited to membrane skeleton organization, metabolic regulation, and mechanisms underlying high deformability.

However, taking human mature red blood cells as a general reference model of red blood cells may ignore another stable circulating cell form in vertebrates [6]. The mature red blood cells of birds, fish, amphibians, and most reptiles retain their nuclei, and in some species, they also retain organelles such as mitochondria, enabling them to perform oxygen transport, metabolic regulation, stress sensing, immune responses, and cell fate regulation [7,8]. A comparative analysis of 513 vertebrate species by Udroiu et al. demonstrated that the mean erythrocyte volume is shaped by multiple evolutionary and physiological factors, including metabolic rate, hemoglobin-oxygen affinity, and genome size in nucleated taxa [9]. Therefore, nucleated red blood cells are not regarded as “unfinished enucleated” red blood cells, but can be understood as another circulating cell with independent biological significance [6,10]. In addition, mechanical differences observed in cross-species comparisons can be interpreted within the context of evolutionary history, ecological adaptation, cell size, nuclear size, and physiological temperature. Therefore, the presence or absence of a nucleus represents one important structural variable, but it is not considered as the sole determinant of erythrocyte mechanical phenotypes.

From the point of view of structure and function, the difference between nucleated red blood cells and human mature red blood cells is not simply to add a nucleus, but to form a multi-level structure system composed of membrane lipid bilayer, transmembrane protein, membrane skeleton, hemoglobin cytoplasm, nucleus/chromatin and organelles [11]. The existence of nucleus not only affects the spatial distribution and geometric shape of cells [12], but also may change the load transfer, local stiffness, mechanical anisotropy and deformation behavior in narrow channels as an internal mechanical unit. At the same time, the preserved organelles and transcription ability enable nucleated red blood cells to actively respond to oxidation, hypoxia, heat, infection and mechanical stimulation [13,14,15]. However, the understanding of how these structural characteristics are translated into mechanical phenotypes and circulatory functions remains incomplete, particularly due to the lack of direct evidence linking the mechanical differences between the nucleus and cytoplasm regions, stress-induced mechanical remodeling, and functional outcomes.

In recent years, with the development of atomic force microscopy (AFM), microfluidic single cell analysis, optical tweezers, confocal imaging and multi-omics, the study of red blood cells is shifting from static morphological description to integrated analysis of structure, mechanics and function [3,16]. On the one hand, the study of human red blood cells has established a mature evaluation system of membrane skeleton structure, oxidative damage and deformability, which can be used as a reference frame to understand the mechanical behavior of red blood cells [16]. On the other hand, studies on nucleated erythrocytes in birds and fish have gradually shown that they have active functions such as transcriptional regulation, pathogen identification, cytokine production, phagocytosis, oxidative response and programmed cell death [17,18,19,20]. The review by Liang et al. on erythrocyte deformability measurement methods further emphasized that different techniques probe erythrocyte mechanics at distinct scales, and that no single parameter is sufficient to comprehensively evaluate microcirculatory function [3]. These findings suggest that nucleated red blood cells may be an important natural model connecting nuclear mechanics, stress biology and circulatory function [17,19,20].

Nucleated red blood cells provide a unique biological model for studying relationships among erythrocyte structure, mechanics and function [21,22,23]. Different from most nucleated cells in monolayer culture, mature nucleated red blood cells are in a suspended circulation environment for a long time, constantly bearing shear force [22], oxidative load [24] and repeated narrow deformation [21,22], while retaining the nucleus and some organelles [23,24]. Therefore, this model can integrate nuclear mechanics, membrane cytoskeleton mechanics, metabolic regulation, and immune response processes that are often studied separately within the same cellular system [21,23,25].

Accordingly, this Review aims to integrate current evidence on relationships among structure, mechanics and function in nucleated erythrocytes and to clarify how nuclear and organelle retention, membrane–skeleton organization, metabolic regulation, and stress responses shape mechanical phenotypes and circulatory function. Human mature red blood cells are used only as an enucleated mechanical reference for conserved membrane–skeleton organization and high deformability, rather than as a replacement model for nucleated erythrocytes. The Review further compares representative vertebrate models, evaluates the interpretive boundaries of commonly used mechanical and imaging approaches, and identifies the key evidence gaps that can be addressed to establish causal links among structure, mechanics, stress responses, and function.

## 2. Methods

This article is a narrative review and is not a systematic review or a scoping review. Literature searches were performed in PubMed, Web of Science Core Collection, Scopus, and Google Scholar for publications available through 12 October 2026, using combinations of terms related to nucleated erythrocytes across vertebrate groups, human red blood cells, erythrocyte mechanics and deformability, AFM and microfluidics, and oxidative, hypoxic, infectious, and immune stress responses. Original research articles, reviews, and methodological studies were selected for their relevance to an approach relating structure, mechanics and function and were synthesized qualitatively. No formal systematic/scoping-review protocol, exhaustive screening procedure, or risk-of-bias assessment was applied. The final synthesis included 128 publications, with quantitative comparisons restricted to datasets with sufficiently comparable methods and experimental conditions.

## 3. Structural Basis and Evolutionary Balance of Nucleated and Enucleated Red Blood Cells

**Figure 1:**
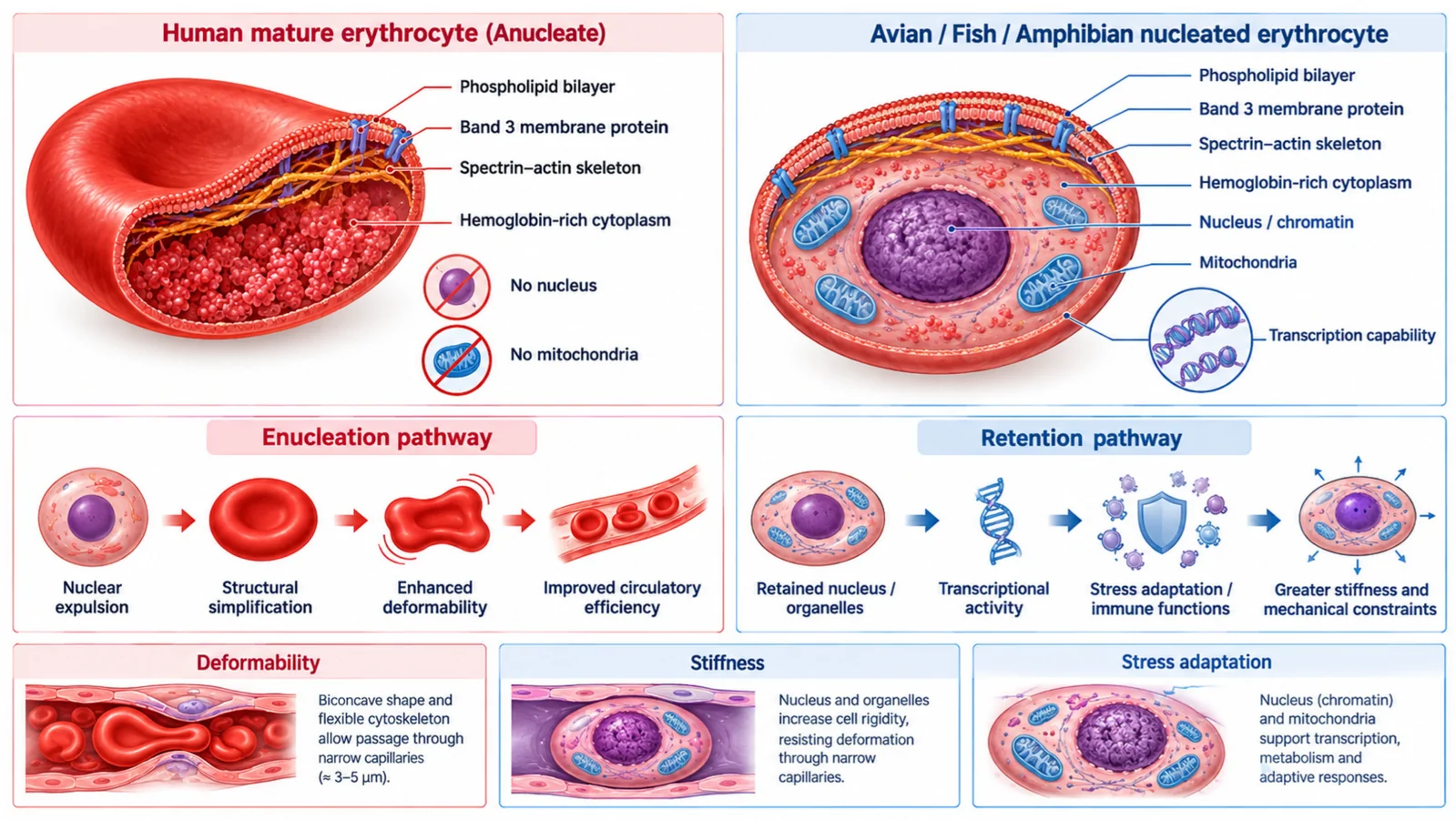
Structural comparison and evolutionary trade-off model between mature mammalian erythrocytes and nucleated erythrocytes.

The distinction between nucleated red blood cells and human mature red blood cells extends beyond the simple classification of “nucleated” versus “enucleated.” It encompasses multiple structural and functional aspects, including overall cell geometry, membrane and membrane skeleton organization, nuclear chromatin features, cytoplasmic hemoglobin content, and organelle composition. The mature red blood cells of birds, fish, amphibians and most reptiles retain their nuclei and organelles such as mitochondria in some species, so they have more complex internal spatial organization. These observable structural variables constitute a comparative baseline to explain the subsequent mechanical, metabolic and stress differences. The structural divergence between mature mammalian erythrocytes and nucleated erythrocytes reflects an evolutionary trade-off between structural simplification for enhanced circulatory efficiency and the retention of cellular complexity for functional versatility (Figure 1). This approach helps distinguish nucleus-associated effects from other species-specific determinants of mechanical behavior.

### 3.1. Differences in Overall Morphology and Size of Cells

Human mature red blood cells typically have a diameter of approximately 7–8 μm and exhibit a biconcave disk shape with a thin center, a thick periphery, and a high surface area-to-volume ratio. Nucleated erythrocytes in most non-mammalian vertebrates are elliptical or oval in shape, with a centrally located protruding nucleus, and are typically larger than their mammalian counterparts [26,27]. Soulsbury et al. found in a cross-species study of birds that red blood cell size and shape are statistically associated with life-history and behavioral traits related to energy demands [28]. A reanalysis by Bury et al. of erythrocytes in squamate reptiles indicated that variations in cell and nuclear morphology are governed by constrained scaling relationships rather than representing independent adaptive modifications [29]. Therefore, when comparing nucleated and enucleated red blood cells, classification need not rely solely on “species” or “nucleated red blood cells” as variables. Instead, geometric parameters such as long axis, short axis, area, thickness, nuclear area, and the nuclear-to-cell area ratio can also be recorded. These scale variables are primarily structural descriptors and also serve as essential prerequisites for subsequent mechanical comparisons. If cell size and nuclear size differ substantially among species, direct comparisons based solely on single stiffness or deformability indices may confound the effects of structural scale with those of the nucleus. Therefore, a more reasonable comparison order is to make clear the distribution of morphology and size within a single species, and then make cross-species comparisons under unified conditions, and take cell size, nuclear size and nucleus-to-cytoplasm ratio as covariables.

### 3.2. Structural Characteristics of Nucleus and Chromatin

The nucleus is the most prominent structural feature distinguishing nucleated red blood cells from human mature red blood cells, but its significance extends beyond simply occupying cytoplasmic space. Sourani et al. demonstrated that chicken erythrocytes retain persistent, experience-dependent DNA methylation patterns associated with early environmental exposures [30]. Combined with evidence of cytokine transcriptional responses during H9N2 infection [31], these findings indicate that mature avian erythrocyte nuclei retain the capacity for stress-responsive gene regulation. Therefore, the nucleus is not only a highly condensed internal structure but also retains the capacity for regulated nuclear responses. Niide et al. found that nuclear area and cell area did not follow identical scaling relationships within or between species. They further observed that nuclear pore complexes and lamins in *Xenopus laevis* red blood cells exhibited sparse and punctate perinuclear distribution patterns [32]. This suggests that nuclear size cannot be simply predicted from cell size using a fixed ratio. For nucleated red blood cells, more informative structural parameters can include nuclear area, nuclear major axis length, nuclear height, nuclear-to-cell area ratio, and chromatin condensation degree [33].

### 3.3. The Common Foundation of Membrane and Membrane Skeleton

Regardless of whether red blood cells retain their nuclei, the cell membrane and underlying cytoskeleton serve as the common structural basis for maintaining cell boundaries and morphological stability. Human red blood cells are the most extensively studied at this level [34]. In human erythrocytes, the membrane skeleton is composed of a highly organized spectrin–actin network. The spectrin–actin junctional complexes are stabilized by associated proteins, including protein 4.1R, adducin, tropomodulin, and dematin [10]. In addition, band 3 represents a major transmembrane component that links the membrane to ankyrin and spectrin, contributing to membrane mechanical stability [1]. Li et al. determined the structure of the native spectrin–actin junctional complex in erythrocytes using cryo-electron microscopy, revealing the spatial organization of short filamentous actin (F-actin) filaments and associated components, including adducin, tropomodulin, dematin, and tropomyosin [10]. Nowak et al. further found that short actin filaments within the membrane skeleton exhibit nanoscale dynamics and are not completely static scaffolds [34]. These results provide a high-resolution reference for understanding the spatial organization of the erythrocyte membrane skeleton.

In contrast, systematic studies of the membrane skeleton composition, junctional density, and spatial organization of non-mammalian nucleated red blood cells at comparable resolution remain limited. Although nucleated erythrocytes also possess spectrin–actin-based membrane skeletons, their molecular composition, junctional density, and quantitative organization remain insufficiently characterized. Current evidence is insufficient to demonstrate that membrane skeleton networks across different vertebrate groups are completely equivalent in terms of nanostructure and mechanical properties.

Beyond these conserved membrane–skeleton components, nucleated erythrocytes possess additional cytoskeletal structures absent from mature mammalian erythrocytes. Avian erythrocytes contain microtubule-based marginal bands that contribute to their elliptical morphology and mechanical stability [35]. These additional cytoskeletal elements may influence cellular deformation and microcirculatory behavior. Consistently, avian nucleated erythrocytes exhibit distinct rheological properties, including differences in the Fahraeus–Lindqvist effect compared with human erythrocytes, suggesting that nuclear retention and cytoskeletal organization contribute to species-specific blood flow characteristics [36].

### 3.4. Organelle Retention and Metabolic Structure Difference

Mature human erythrocytes lack nuclei and most organelles, with metabolism primarily dependent on pre-existing enzyme systems [37,38]. In contrast, nucleated erythrocytes retain more complete intracellular structures, and functional mitochondria are preserved in some birds and fish [39,40]. Bhardwaj et al. observed that mitochondrial membrane potential, reactive oxygen species (ROS) levels, and the expression of genes involved in antioxidation and lipid metabolism in red blood cells changed synergistically in response to the simulated migratory state. These findings indicated that mature avian red blood cells did not rely solely on pre-stored proteins to maintain homeostasis but instead possessed active regulatory capacity supported by organelles and transcriptional systems [39]. In human mature red blood cells, research has focused more on how to maintain the stability of existing membrane proteins, metabolic enzymes, and antioxidant systems [37,38]. For nucleated red blood cells, in addition to membrane and hemoglobin maintenance, the regulatory capacity provided by mitochondria, nuclear transcription, and protein renewal can also be considered [39,40,41].

### 3.5. Evolutionary Trade-Off Between Denucleation and Nuclear Preservation

Erythroid enucleation in mammals is an active remodeling process involving nuclear polarization, cytoskeletal reorganization, nuclear condensation, and nuclear extrusion during terminal differentiation [11]. Studies on PIM1 and AURKA further indicate that kinase-regulated cytoskeletal remodeling and vesicular trafficking contribute to this process [12,42]. From an evolutionary perspective, enucleation reduces intracellular complexity and creates a highly deformable erythrocyte optimized for circulation, but it also limits transcriptional activity and organelle-dependent renewal capacity. In contrast, non-mammalian nucleated erythrocytes retain nuclear and organelle-associated functions, representing a structurally more complex cellular strategy. Comparative studies across vertebrates suggest that erythrocyte size and morphology are shaped by metabolic demands, oxygen transport characteristics, and ecological adaptation [9,28]. Therefore, human mature erythrocytes can serve as a simplified mechanical reference, whereas nucleated erythrocytes provide models for studying integrated nuclear, cytoplasmic, and organelle-associated regulation. The structural differences among representative erythrocyte models are summarized in Table 1.

## 4. Relationships Among Structure, Mechanics, and Function: The Core Biophysical Model of Nucleated Red Blood Cells

**Figure 2:**
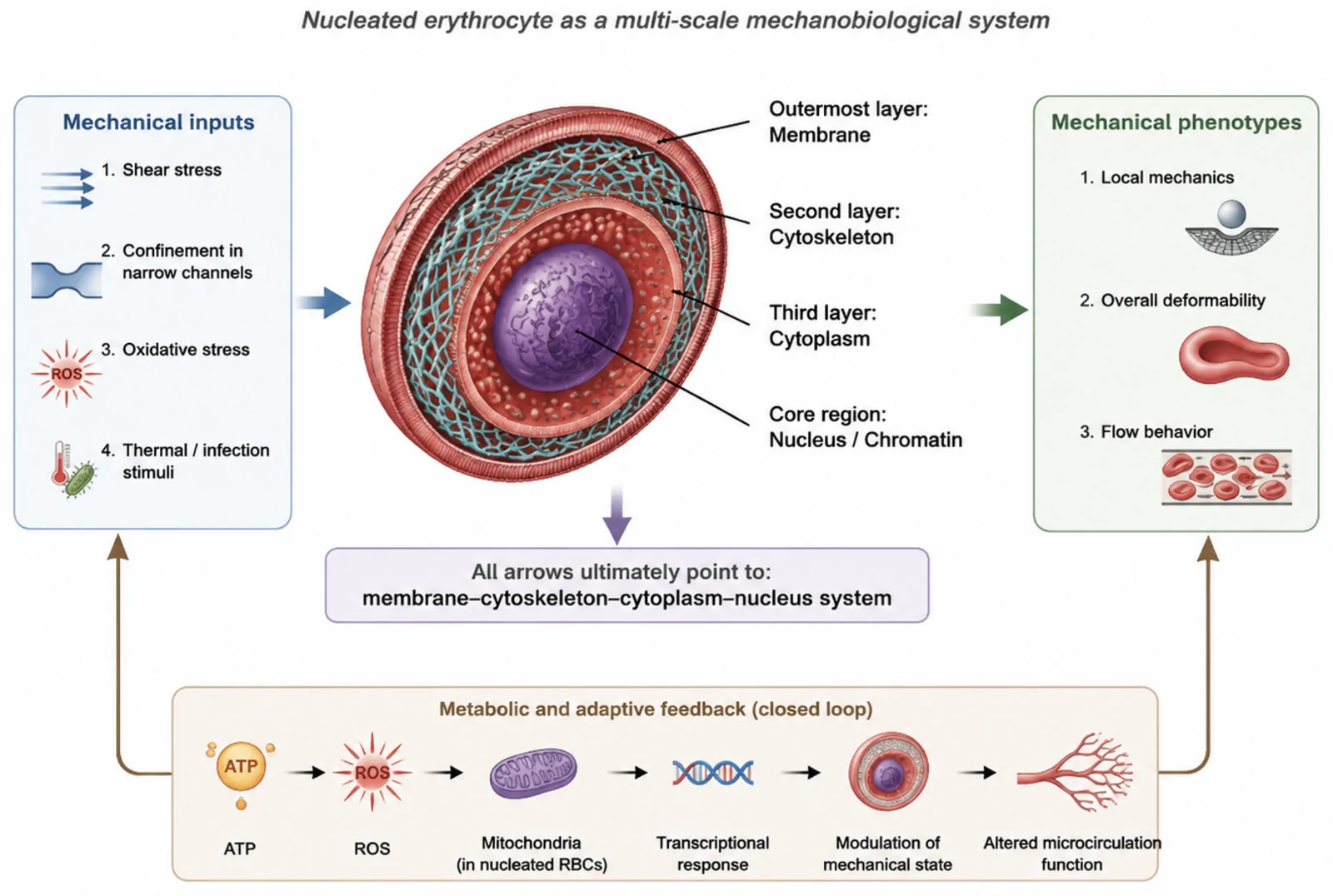
Model linking structure, mechanics, and function across the membrane–cytoskeleton–cytoplasm–nucleus system in nucleated erythrocytes.

The mechanical behavior of nucleated red blood cells is determined not by a single structure, but by the synergistic effects of multiple structural levels, including the membrane, membrane skeleton, cytoplasm, and nucleus. This integrated mechanical property ultimately underpins their circulatory function. External shear, compression and stenosis first act on the cell membrane and membrane skeleton, and then transfer to the inside of the cell through cytoplasmic pressure, viscous flow and cell volume change [43]. In this process, hemoglobin concentration, oxygenation state, and osmotic state jointly regulate cytoplasmic viscosity and overall deformation. Meanwhile, the nucleus and chromatin constitute unique internal structural constraints in nucleated red blood cells, potentially leading to mechanical heterogeneity and anisotropic behavior between the nucleus and cytoplasm. At the same time, adenosine triphosphate (ATP) supply and redox state can provide feedback regulation of membrane skeleton stability, cytoplasmic properties, and cell volume, causing the mechanical properties of red blood cells to exhibit dynamic changes with cellular state rather than fixed material characteristics. The above-mentioned changes in local structure and single cell mechanics can only be truly transformed into functional consequences at the circulatory level if they further affect the passage of narrow channels, microcirculatory distribution, population rheology or circulatory survival [44]. This hierarchical relationship among cellular structures, mechanical phenotypes, and circulatory function is summarized in Figure 2, which illustrates a model linking structure, mechanics, and function across the membrane–cytoskeleton–cytoplasm–nucleus system in nucleated erythrocytes.

Focusing on how local structure and mechanics relate to whole-cell function, this paper discusses several key aspects. These include the cooperative deformation of the membrane–cytoskeleton–cytoplasm system, the internal mechanical constraints between the nucleus and cytoplasm, the dynamic regulation of metabolic and redox states on cellular mechanical phenotypes, and the scale transition from single-cell deformation to circulatory function. Human mature red blood cells are used as a mechanical reference for an enucleated and highly deformable system, allowing the shared mechanisms of red blood cells, the specific mechanisms of nucleated red blood cells, and the inferences that remain primarily based on mechanobiological principles to be distinguished.

### 4.1. Membrane–Skeleton–Cytoplasm Coupling: The Basis of Overall Deformability

When red blood cells are subjected to external loads, the first response is the cell membrane and its underlying membrane skeleton. Studies on human mature red blood cells have shown that lipid bilayer mainly provides bending resistance and barrier function, while in-plane shear elasticity is mainly borne by spectrin–actin network. The vertical connection between Band 3 and ankyrin and 4.1R protein connects transmembrane protein with membrane skeleton [10]. Issaian et al. further identified the oxygen-dependent regulatory axis of Band 3–biliverdin reductase B (BLVRB) through the deep protein omics and protein interactive omics research, and linked the oxygen-dependent protein interaction at the N-terminal of Band 3 with glycolysis and redox remodeling [45]. Therefore, the membrane-skeleton is not an isolated mechanical shell, but an interface connecting ion exchange, metabolic distribution and structural stability at the same time. For nucleated red blood cells, although direct evidence for a fine membrane–skeleton network structure comparable to that of human red blood cells remains limited, it is reasonable to consider membrane lipids, transmembrane proteins, and the cytoskeleton as the first mechanical layer through which external loads are transmitted into the cell.

The overall deformability cannot be explained by the mechanical properties of the membrane-skeleton alone [46]. Hemoglobin is the main macromolecular component of cytoplasm, and its concentration, oxygenation state and cell water content jointly affect the viscosity and volume of cytoplasm [47]. In a randomized, double-blind, crossover study, Chatzinikolaou et al. demonstrated that exercise and oxidative stress dynamically modulate erythrocyte glycolysis and redox metabolism, thereby reshaping metabolic flux distribution toward ATP, nicotinamide adenine dinucleotide phosphate (NADPH), and 2,3-bisphosphoglycerate (2,3-BPG) production [48]. Insufficient ATP can affect ion pump activity and cell volume regulation, and the change in osmotic state will change both membrane tension and cytoplasmic concentration [49]. It can be seen that the local apparent stiffness, overall deformation and group deformability measured by AFM reflect different levels of mechanical properties. If the membrane–skeleton state, cytoplasmic viscosity, and cell volume are not considered simultaneously, reductions in overall deformation caused by cytoplasmic or osmotic changes may be incorrectly attributed to “increased membrane–skeleton stiffness.”

### 4.2. Nuclear–Cytoplasmic Coupling: Internal Constraints and Mechanical Anisotropy

The key mechanical difference between nucleated red blood cells and human mature red blood cells lies in the existence of nuclei inside. The nucleus is typically located at the center of the cell, and the highly condensed chromatin within it results in pronounced spatial heterogeneity in cell thickness, mass distribution, and intracellular viscoelasticity. Niide et al. found that in amphibian erythrocytes, nuclear size does not scale linearly with cell size. This implies that nuclear area, nuclear thickness, and the nucleus-to-cell ratio may each independently influence the mechanical phenotype [32]. For cells passing through narrow spaces, membrane unfolding and cytoplasmic rearrangement can accommodate part of the imposed deformation. However, when the constriction size approaches the dimension of the nucleus, nuclear compression, rotation, and reorientation are more likely to introduce additional deformation resistance. Therefore, nucleated red blood cells are more appropriately regarded as composite systems consisting of a deformable membrane–skeleton, viscous cytoplasm, and viscoelastic nucleus, rather than being directly described using the homogeneous thin-shell approximation applied to human mature red blood cells.

Direct mechanical measurements support a contribution of the nucleus to erythrocyte mechanics, but they do not indicate a universal increase in whole-cell stiffness. In in-vitro human erythroid cells, Guzniczak et al. reported a mean Young’s modulus of 1.04 ± 0.47 kPa for nucleated cells compared with 0.53 ± 0.12 kPa after enucleation, while isolated nuclei exhibited a substantially higher modulus of 7.06 ± 4.07 kPa [50]. Thus, within this maturation system, nuclear retention was associated with an approximately twofold increase in cellular stiffness. In contrast, spatially resolved measurements in mature chicken erythrocytes revealed strong intracytoplasmic heterogeneity. Baier et al. measured median apparent Young’s moduli of 2278 Pa above the nucleus and 461 Pa in the peripheral region at 25 °C; at 42 °C, the corresponding values were 2384 and 183 Pa [51]. Notably, the peripheral modulus at 42 °C was close to the 179 Pa reported for adult human erythrocytes under comparable saline conditions. The differences between these studies likely reflect both biological and methodological factors, including species, maturation state, cell and nuclear geometry, temperature, medium, and indentation location. Thus, the nucleus appears to contribute to local mechanical heterogeneity, whereas its effect on whole-cell stiffness is context dependent rather than universal.

However, when comparing different vertebrate species, the contribution of the nucleus can be evaluated together with differences in cellular geometry, nuclear-to-cytoplasmic ratio, metabolic state, and thermal environment, because these factors may jointly influence the measured mechanical phenotype. At present, there is no evidence that the red blood cells of mature birds or fish have the same direct cytoskeleton–nucleus mechanical force transmission as typical adherent cells, so direct molecular connection is not described as an established mechanism [52]. A more reliable explanation is that external forces first alter the overall geometric configuration of the membrane and cell. Subsequently, the load is transmitted to the nucleus through cytoplasmic pressure, viscous flow, intracellular crowding effects, and the perinuclear space. The size, position, and mechanical properties of the nucleus then impose constraints on the overall deformation [53]. Because most nucleated red blood cells and their nuclei are oval-shaped, loading along the long and short axes may produce different equivalent mechanical responses. Moreover, the angle between the nuclear long axis and the flow direction may also influence the resistance to passage through narrow channels.

At present, these judgments about nuclear size, orientation and mechanical anisotropy are mainly based on structural characteristics and general mechanical biology principles, and still can to be directly verified by AFM spatial mapping of living cells, synchronous nuclear imaging and microfluidic confined experiments. In particular, it is important to distinguish between the concepts of “the nuclear region being measured as stiffer” and “the nucleus itself being intrinsically stiffer”, because local thickness, membrane tension, cytoplasm crowding and basal effect may all lead to the increase in apparent modulus. Only after the spatial registration of nuclear region and cytoplasmic region is completed in the same cell and the depth of indentation is controlled, can the independent contribution of nucleus to the overall mechanical heterogeneity be estimated more reliably.

### 4.3. Metabolism–Redox Feedback: Dynamic Adjustment of Mechanical State

The mechanical state of red blood cells is not a fixed material parameter, but will change with metabolism and redox environment [54]. Although human mature red blood cells lack nuclei and mitochondria, they still rely on complex redox networks to maintain membrane, hemoglobin, and cytoskeletal stability [55]. Using a multi-omics approach, Keele et al. demonstrated that alteration of β-globin Cys93 (β-Cys93) disrupts glutathione redox homeostasis and alters multiple redox-related post-translational modifications [56]. Turcitu et al. further found that oxidative stress induced by diamide can produce dose-dependent and non-monotonic changes in erythrocyte morphology and deformability [57]. Therefore, in the approach relating structure to mechanics, ROS, ATP, hemoglobin oxidation state and membrane skeleton integrity are not independent indicators, but important state variables that jointly determine the mechanical phenotype.

Unlike human mature erythrocytes, nucleated red blood cells retain mitochondria and transcriptional activity, potentially providing an additional regulatory layer for metabolic adaptation and stress responses [39]. Through mitochondrial function and gene regulation, nucleated erythrocytes may modulate ROS production, energy supply, and antioxidant responses in ways that are unavailable to mature mammalian erythrocytes [40]. Current evidence from avian erythrocytes suggests that mitochondrial activity can change under physiological conditions, but the functional consequences remain incompletely quantified. Bhardwaj et al. reported increased mitochondrial membrane potential (MMP), reduced ROS accumulation, and altered expression of antioxidant and lipid-metabolism genes in red-headed bunting erythrocytes during simulated migration [39]. However, these measurements did not directly quantify mitochondrial contribution to oxidative phosphorylation, glycolytic flux, pentose phosphate pathway (PPP) activity, or 2,3-BPG regulation. Therefore, mitochondrial potential alone cannot determine how retained mitochondria alter erythrocyte energy metabolism, hemoglobin oxygen affinity, or mechanical properties.

Comparison with human erythrocytes further highlights the current quantitative gap. Human mature erythrocytes lack mitochondria and mainly rely on anaerobic glycolysis, but their metabolic flux distribution has been quantitatively characterized. Oxley et al. used ^13^C-label tracing combined with multinuclear nuclear magnetic resonance (NMR) analysis and demonstrated that approximately 27% of glycolytic flux enters the Rapoport–Luebering shunt for 2,3-BPG production and approximately 10% enters the PPP [58]. These quantitative parameters directly link glucose metabolism with oxygen-affinity regulation and antioxidant capacity. In contrast, comparable measurements separating glycolysis and oxidative phosphorylation in avian nucleated erythrocytes remain largely unavailable, limiting mechanistic interpretation of their metabolic advantages. Furthermore, seasonal studies indicate that mitochondrial retention in avian erythrocytes does not necessarily correspond to uniformly increased oxidative phosphorylation. Nord et al. measured seasonal differences in mitochondrial volume and respiratory activity in erythrocytes from three tit species [59]. Their results showed that seasonal adaptation involved changes in mitochondrial volume, respiratory activity, and uncoupling rather than simply enhanced ATP-producing oxidative phosphorylation.

A rigorous evaluation of metabolism–redox feedback in nucleated erythrocytes requires integrated measurements of mitochondrial function, metabolic flux, redox regulation, oxygen affinity, and deformability under standardized conditions. Future comparative studies combining metabolic flux analysis, mitochondrial functional assays, and oxygen-transport measurements will help to determine how retained organelles modify erythrocyte physiology and mechanical behavior.

### 4.4. From Single Cell Deformation to Circulatory Function: Scale Integration and Reference Boundary of Human Red Blood Cells

Structural changes can only be further transformed into circulatory function when they change the overall deformation, microvascular passage, circulatory retention or group rheology of cells. Ebrahimi and Bagchi computational studies show that the decrease in erythrocyte deformability can alter the distribution of red blood cells and local hematocrit in bifurcated microvascular networks, indicating that the mechanical properties of individual cells can be amplified in blood flow networks [60]. Islamzada et al. further proved in the in vivo infusion model of mice that the erythrocyte subsets with obviously low deformability distribution were selectively eliminated within 24 h, which provided new in vivo evidence for the relationship between mechanical phenotype and circulatory fate [44]. However, this scale-up is not linear, and hematocrit, plasma viscosity, cell aggregation, cell collision, microstructure and geometric constraints of blood vessels and spleen will all affect the final microcirculation perfusion and cell clearance [61]. Therefore, for nucleated red blood cells, a more reasonable interpretation is that changes in local structure or material properties first affect the deformability and post-deformation recovery of individual cells. These alterations may subsequently influence population-level rheological properties, microcirculatory distribution, or cell clearance processes, ultimately affecting gas transport function. This hierarchical distinction also shows that local AFM parameters can be interpreted in combination with microfluidic throughput, recovery kinetics or related endpoints in vivo circulation [62].

The research approach of membrane skeleton, metabolism and deformability is the most mature, which can be used to define the sharing index and serve as an important reference for evaluating the mechanical properties of highly flexible cell systems after enucleation [63]. However, it lacks nuclear, mitochondrial and newborn transcription reactions, and cannot directly represent the internal mechanical constraints and active regulation mechanism of nucleated red blood cells. Therefore, Band 3, spectrin/actin, ATP, cell volume, deformability and redox state can be taken as the shared indexes for cross-group comparison, and the nuclear size, nuclear orientation, chromatin state and organelle reaction can be analyzed separately as the specific modules of nucleated red blood cells [64]. This stratification can avoid conflating the mechanisms shared by red blood cells with those specific to nucleated cells, while ensuring that subsequent stress responses are directly linked to measurable structural and mechanical variables. To further clarify the relationship among stress input, mechanical response, and functional outcome, representative experimental evidence from chicken, fish, and human erythrocytes is summarized in Table 2. Accordingly, the comparison model distinguishes shared mechanical principles derived from human erythrocytes from the unique biological properties of nucleated erythrocytes, including nuclear regulation, organelle-associated responses, and active stress adaptation.

## 5. Stress Response: From Structural Disturbance to Mechanical Remodeling and Functional Consequences

**Figure 3:**
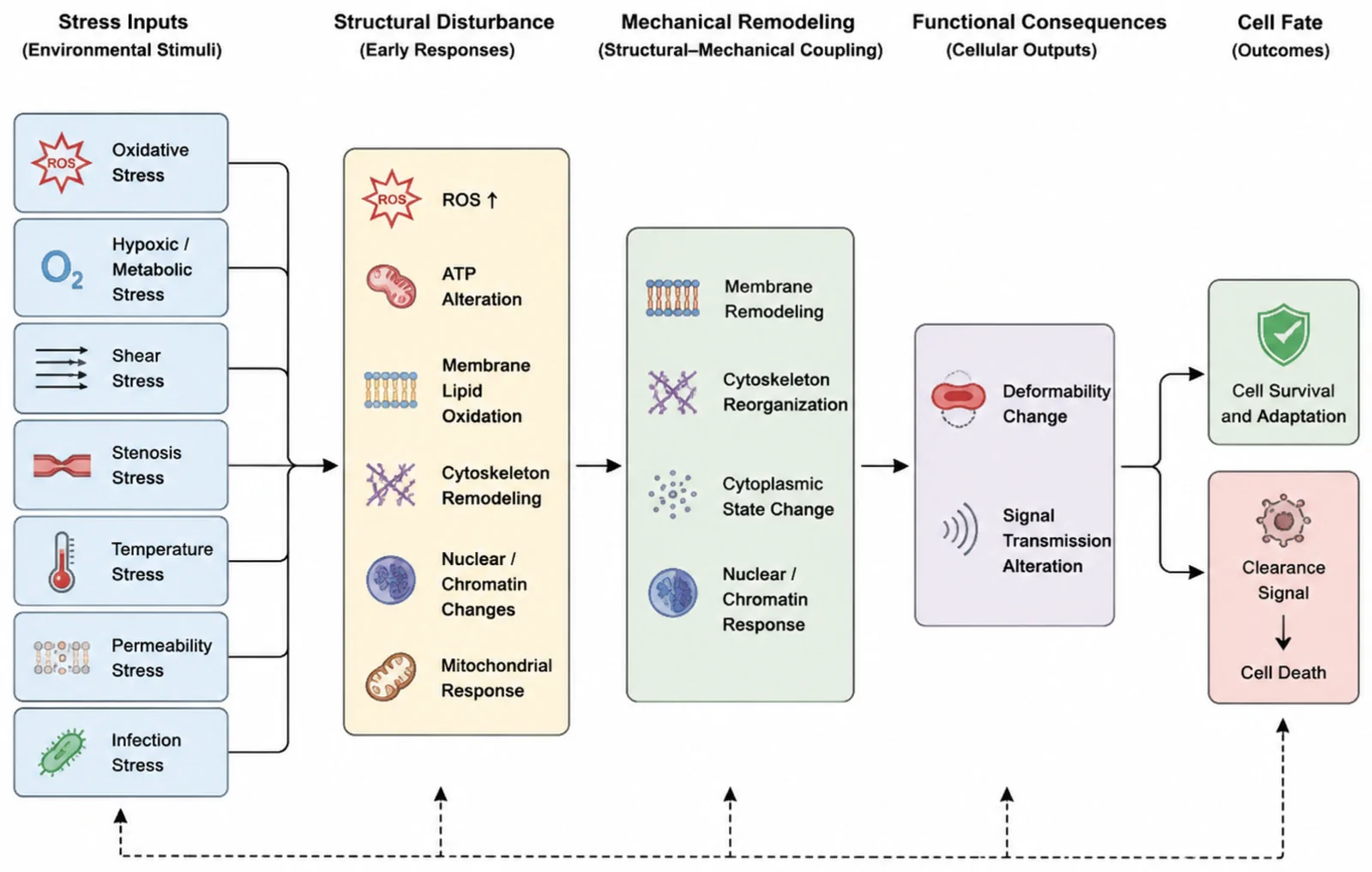
Stress-response network linking environmental stimuli to mechanical remodeling and cell fate. Solid arrows indicate the proposed progression of cellular responses through successive stages, whereas the dashed line and upward arrows indicate potential interactions among different stages.

Stress conditions provide a context for evaluating how structural alterations are translated into mechanical remodeling and functional consequences. Rather than considering individual stressors separately, this section integrates oxidative, metabolic, mechanical, thermal, and infection-related challenges within the framework of stress input–structural disturbance–mechanical response–functional outcome. The relationship between stress exposure, cellular remodeling, and functional consequences is summarized in Figure 3. In this section, stress is used to denote erythrocyte-level stress stimuli and the resulting cellular responses, including mechanical, oxidative, hypoxic, thermal, toxic, and infection-related challenges. It does not refer to organismal psychological stress or nonspecific vascular stress.

### 5.1. Oxidation and Hypoxia/Metabolic Stress: Intracellular Steady State Imbalance and Structural–Mechanical Remodeling

Oxidative and metabolic stress provides a clear example linking intracellular state changes with erythrocyte mechanical remodeling [69,70]. In human mature red blood cells, oxidative imbalance involving hemoglobin oxidation, free heme/iron accumulation, and altered glycolytic regulation can promote membrane damage, Band 3 modification, spectrin disruption, and reduced deformability [65,69,71]. These findings establish the pathway of “metabolic/redox disturbance–membrane skeleton remodeling–mechanical impairment” as a reference framework. Nucleated erythrocytes may possess additional regulatory layers through mitochondrial activity and nuclear transcription. In avian erythrocytes, metabolic state changes have been associated with alterations in ROS regulation, mitochondrial membrane potential, antioxidant responses, and stress-related gene expression [39]. Dietary and physiological adaptations may further influence erythrocyte metabolic and structural plasticity [72,73]. Therefore, oxidative stress in nucleated erythrocytes may not directly translate into equivalent mechanical damage, but instead depends on the balance between structural injury and active adaptation. However, direct evidence linking these responses to local mechanics or microcirculatory function remains limited.

### 5.2. Mechanical, Thermal and Osmotic Stress: Deformation Load and Structural Reversibility Driven by Physical Environment

Shear and stenosis deformation in circulation are the basic mechanical loads that red blood cells continue to bear. The calculation of three-dimensional microvascular network by Ebrahimi and Bagchi shows that the decrease in erythrocyte deformability can change the distribution of cells at bifurcation, local hematocrit, microvascular perfusion and vascular resistance, suggesting that the mechanical state of single cells can be amplified in the microcirculation network [60]. For nucleated red blood cells, the internal geometric constraints are added to the central nucleus. When the external constraints are close to the nuclear size, in addition to membrane unfolding and cytoplasmic rearrangement, nuclear rotation, orientation change and stress concentration around the nucleus may occur [51]. It can be emphasized that this nuclear confinement mechanism represents a biophysically plausible interpretation based on current structural and mechanical principles; however, direct high-speed imaging and quantitative mechanical evidence remain limited, and this mechanism should not be considered a definitive conclusion applicable to all species. Greene et al. compared stress-related gene expression and mechanical properties of erythrocytes from different broiler strains and red junglefowl, and found that cellular mechanical phenotypes varied with genetic background and heat stress conditions. These findings provided direct evidence for coordinated alterations between molecular stress responses and cellular mechanical properties in poultry [66].

Heat and osmotic changes can also directly change the physical microenvironment of red blood cells. Greene et al. found that the expression of heat shock protein (HSP), cytokine and chemokine-related genes changed synchronously with the mechanical properties in chicken erythrocytes under short-term heat stress at 45 °C, which is one of the direct evidences connecting the thermal response and mechanical phenotype of avian erythrocytes [66]. Fregin et al. investigated the effects of temperature on red blood cell mechanics through single-cell rheology and found that both human and bat red blood cells transitioned toward a more viscous mechanical state as the temperature decreased. This finding indicates that temperature itself can reshape the time-dependent mechanical response of red blood cells. However, these results cannot directly substitute for direct evidence obtained from nucleated erythrocytes within the same avian species [74]. Thermal stimulation may affect membrane fluidity, protein conformation, mitochondrial ROS and nuclear chromatin state at the same time, while osmotic changes directly change cell water content, volume and membrane tension. For nucleated red blood cells, the volume change will also readjust the spatial ratio between nucleus and cytoplasm, so that the same cell has different internal mechanical constraints at different temperatures or osmotic conditions. Therefore, the key to this kind of physical stress lies not only in whether the cell is deformed, but also in whether its morphology and mechanical state can be restored after the external stimulus is lifted. Reversible volume and mechanical changes are more consistent with adaptive adjustment, whereas persistent membrane skeleton abnormalities, nuclear structural changes, or delayed recovery are more likely to indicate cumulative injury.

### 5.3. Infection-Related Stress and Immune Response: Functional Activation and Its Potential Mechanical Cost

Infection studies have provided key evidence for redefining the functional role of nucleated red blood cells [75]. Avian erythrocytes can actively participate in pathogen recognition, cytokine and antimicrobial peptide production, ROS regulation, immune adhesion, phagocytosis and apoptosis [17]. Studies showed that avian pathogens such as *Escherichia coli* and H9N2 virus can induce immune-related responses in chicken erythrocytes, while goose erythrocytes exhibit antibacterial activity associated with phagocytosis and respiratory burst reactions [76]. Fish erythrocytes further support this concept, with carp erythrocytes showing increased inflammatory cytokine expression, ROS production, phagocytosis and *tlr4*/*tlr9* activation after *Aeromonas hydrophila* infection [41]. In Atlantic salmon, viral infection can modify erythrocyte surface properties and alter recognition processes [67]. These findings indicate that nucleated red blood cells possess pathogen- and species-specific immune functions beyond oxygen transport, although their responses vary among different host–pathogen interactions.

Importantly, phagocytic activity is not consistently detected across teleost erythrocyte models. Campos-Sánchez et al. reported that erythrocytes isolated from gilthead seabream (*Sparus aurata*) and European sea bass (*Dicentrarchus labrax*) showed no detectable phagocytic ability or respiratory burst after 20–120 min exposure to formalin-inactivated bacteria or heat-killed yeast. Fluorescence microscopy mainly showed target adhesion to the erythrocyte surface, although transmission electron microscopy revealed pseudopod-like membrane folds and cytoplasmic vesicles [77]. This contrasts with common carp (*Cyprinus carpio*), in which internalization of carboxylated latex beads was supported by confocal microscopy, transmission electron microscopy, and serial block-face scanning electron microscopy [41]. Positive phagocytosis-associated antibacterial activity has also been reported in grass carp (*Ctenopharyngodon idella*) and *Clarias fuscus* erythrocytes exposed to bacterial targets or latex particles [78]. The differing outcomes may therefore reflect both genuine species-specific variation and differences in experimental design, including the nature, size, and surface chemistry of the target particles, the use of live, killed, or synthetic targets, incubation time and temperature, erythrocyte isolation and purity, and the imaging or analytical criteria used to distinguish surface adhesion from true intracellular uptake. Thus, phagocytosis can be regarded as a context-dependent erythrocyte capability demonstrated in some teleost species rather than a universal property of fish erythrocytes.

From the perspective of relationships between structure and mechanics, the outstanding deficiency of current infection-related research is not whether red blood cells participate in immune response, but the lack of systematic quantification of the influence of immune activation on their circulatory function and mechanical cost. toll-like receptor (TLR)-mediated signaling pathways, ROS release, membrane receptor aggregation, phagocytosis, or pathogen adhesion, together with subsequent alterations in the cytoskeleton and cell volume, may influence membrane tension, adhesion properties, and deformability [79]. However, most infection-related studies in poultry and fish still focus on quantitative polymerase chain reaction (qPCR), cytokine profiling, and microscopic morphology, while simultaneous measurements of AFM-based local mechanics and microfluidic deformability remain limited. Therefore, whether enhanced immune function occurs at the expense of mechanical stability can be considered a hypothesis linking structure and function that requires further verification, rather than a definitive conclusion.

### 5.4. Combined Stress Effects and Cell Fate: From Adaptive Remodeling to Irreversible Damage

Stress in the real circulation environment is usually not a single factor. Oxidative load can weaken the mechanical stability of membrane lipid and membrane skeleton, making it more difficult for cells to tolerate subsequent shear, and repeated deformation may amplify the existing membrane protein connection defects [80]. The significance of the RoxyScan-based study by Larkin et al. lies in the simultaneous application of oxidant and shear stresses, combined with continuous tracking of deformation changes, demonstrating that erythrocyte responses to combined stresses cannot be simply predicted by summing the effects of individual factors [54]. For nucleated red blood cells, these interactions may also extend to mitochondria and nuclei. Metabolic state can influence ROS production and energy supply, whereas nuclear and chromatin states may alter intracellular mechanical constraints and subsequent transcriptional adaptation. Similar interactions may also occur between infection and shear, heat and oxidation, or hypoxia and mechanical load, but the studies on nucleated red blood cells that directly verify these interactions is still insufficient. Therefore, it is safer to draw a conclusion that compound stress is an important level connecting the single factor phenotype and the real cycle load in the laboratory, and its direction and intensity still can be determined by the experimental evidence within the species.

The final result of stress also involves the fate of cells, and it is especially necessary to distinguish nucleated and enucleated red blood cells at this level. In the past, human mature red blood cells have been used to describe regulatory death-like processes such as Ca^2+^ elevation, cell shrinkage and phosphatidylserine exposure in the model of eryptosis [81]. Chen et al. reported an NLR family pyrin domain-containing protein 3 (NLRP3)-related lytic programmed cell death process in Cell, suggesting that the death spectrum of enucleated erythrocytes need not be summarized solely as eryptosis [68]. Fish and poultry nucleated red blood cells retain a more complete mitochondrial and transcription system, which can show a variety of programmed cell death-related characteristics. Tao et al. observed that multiple programmed cell death pathways were activated following *Aeromonas hydrophila* infection in *Clarias fuscus* erythrocytes, revealing a link between oxidative stress and ferroptotic responses [82]. Therefore, the death types of red blood cells in different species can be judged by combining lipid peroxidation, iron metabolism, mitochondria, caspase, membrane integrity and pathway-specific molecular evidence, rather than simply applying a single death mode based on morphology or directly.

From a cross-species comparison and mechanobiological perspective, a more explanatory approach would be to view stress outcomes as a continuum rather than survival or death as the sole endpoint. This continuum can be divided into four stages: adaptation, mechanical subdamage, signal clearance, and irreversible death, and is distinguished by indicators such as deformability and its resilience, adhesion properties or phosphatidylserine externalization, and membrane integrity [83]. This model can provide a unified scale for the comparison of stress outcomes in different species, and more completely reflect the gradual process of cell adaptation to irreversible damage. On this basis, Table 3 further summarizes the representative research evidence of nucleated erythrocytes in different species, different stress types and functional backgrounds in recent years to show their stress response characteristics and potential functional associations.

## 6. Evidence Integration of Representative Models: Reference of Birds, Fish, Amphibians/Reptiles and Human Erythrocytes

Different models exhibit different levels of evidential maturity across four dimensions: structure, function, mechanics, and stress response. In general, the evidence of avian erythrocytes, especially chicken erythrocytes, is relatively complete in nuclear structure, transcription and heat/infection stress. Fish have made rapid progress in immunity, infection and programmed cell death. Amphibians and reptiles have mainly contributed to investigations at the cellular and nuclear scales. Human mature red blood cells provide a mechanical reference for understanding membrane–skeleton properties, oxidative damage, and deformability. Therefore, different models are more suitable as complementary evidence sources, rather than replacing each other.

### 6.1. Avian Red Blood Cells: A Comprehensive Model with the Most Complete Multilayer Evidence

Among avian erythrocytes, chicken erythrocytes have been the most extensively studied, with the most established research foundation [84]. The nuclei of chicken red blood cells are highly condensed, but longitudinal methylation studies show that their epigenetic status can change continuously with early environmental experience [30], and infection models also prove that they can produce new immune transcription responses [85]. Liu et al. also showed that pathogen adhesion can be accompanied by time-dependent changes in complement-related genes [18]. At the same time, avian red blood cells can participate in immune processes such as pathogen identification, cytokine and antibacterial molecule production, ROS regulation [17]. Greene et al. simultaneously observed the changes in heat stress-related genes and mechanical properties in different chicken strains [66]. Other bird studies have also shown that the mitochondrial state, ROS and antioxidant/lipid metabolism related genes of red blood cells can change with metabolic needs. Therefore, the poultry model is more suitable to comprehensively explore the correlation mechanism between nuclear structure, metabolic regulation, stress response and mechanical phenotype.

A limitation is that multilevel evidence is still derived from separate studies, while investigations that directly and simultaneously measure the mechanical properties of nuclear and cytoplasmic regions, membrane–cytoskeleton remodeling, and microvascular passage capacity remain limited. Therefore, chicken red blood cells represent a suitable model for establishing an integrated multilevel evidence approach. However, at the current stage, nuclear alterations and transcriptional changes are not considered direct causal drivers of mechanical abnormalities [53].

### 6.2. Fish Red Blood Cells: A Dominant Model for the Study of Immunity, Infection and Cell Fate

Fish mature red blood cells have nuclei and maintain transcription activity, which is one of the most abundant groups in the immune research of nucleated red blood cells [86]. *Cyprinus carpio* erythrocytes have obtained multiple layers of direct evidence such as cytokine secretion, TLR signaling, ROS production, and phagocytic activity [41]. Studies on Atlantic salmon show that virus particles can continuously interact with the surface of red blood cells with sugar chain remodeling, and different salmon hosts also show specific antiviral transcription and apoptosis reactions [19]. In addition, Tao et al. reported that infection induced multiple programmed cell death pathways and ferroptosis-related alterations in *Clarias fuscus* erythrocytes [82]. Therefore, the advantage of fish model is that it can connect pathogen identification, ROS, membrane surface changes, transcription reaction and cell fate. The key gap still lies in whether immune activation, virus binding or death pathway initiation will simultaneously lead to deformation reduction, adhesion enhancement and hemorheological changes. At present, the quantitative relationships among immune function, mechanical phenotype and circulation cost remains to be verified.

### 6.3. Amphibian and Reptile Erythrocytes: An Important Supplement to Nuclear Scale and Comparative Mechanics

Amphibian and reptilian red blood cells usually have large cells and nuclei, which are suitable for studying cell scale, nuclear scale and their proportional relationship. Niide et al. found that the nuclear size and cell size of erythrocytes of Anura do not follow exactly the same scale relationship within and between species [32]. In squamates, Bury demonstrated that cell and nuclear morphologies scale isometrically with each other, rather than independently [29]. These models are therefore helpful to analyze the relationship between nuclear size, cell geometric characteristics and local mechanical properties, and avoid simply attributing the size effect to the nucleated characteristics themselves [9].

However, these models are still mainly supported by comparative evidence from structural and evolutionary perspectives, while studies on mechanical stress and direct mechanical characterization remain limited compared with those in poultry and fish [26]. In addition, the environmental temperature and osmotic state in ectotherms can significantly affect the cell volume, membrane tension and nuclear/cytoplasmic spatial relationship, and the experimental conditions themselves can easily become an important confounding factor [74]. Therefore, amphibian and reptilian models are more suitable as supplementary evidence for nuclear scaling and cross-species mechanical hypotheses, rather than serving as a universal explanation for stress-response mechanisms in nucleated red blood cells.

### 6.4. Cross-Model Integration: Human Red Blood Cell Reference, Sharing Module and Model Selection

Human mature red blood cells should be considered a mechanical reference rather than a mechanistic substitute for nucleated erythrocytes. Their membrane–skeleton organization, oxidative regulation, and deformability have been extensively characterized, providing shared mechanical benchmarks through approaches such as high-resolution structural analysis, oxidation–shear assays, and microfluidic evaluation [87]. However, the absence of nuclei, mitochondria, and active transcription limits their ability to represent nuclear constraints and adaptive responses. Therefore, cross-model comparisons should distinguish conserved erythrocyte features, including membrane components, hemoglobin, volume regulation, and redox stability, from nucleated-cell-specific modules involving chromatin organization, organelles, and transcriptional regulation [88]. Although human erythrocytes also retain certain immune-related functions, such as bacterial DNA sensing through through toll-like receptor 9 (TLR9), nucleated erythrocytes may possess broader and more sustained adaptive responses through active gene regulation [89]. Thus, model selection should be guided by the biological question rather than species classification.

## 7. Multi-Scale Research Methods: Linking Structure, Mechanics, and Function

**Figure 4:**
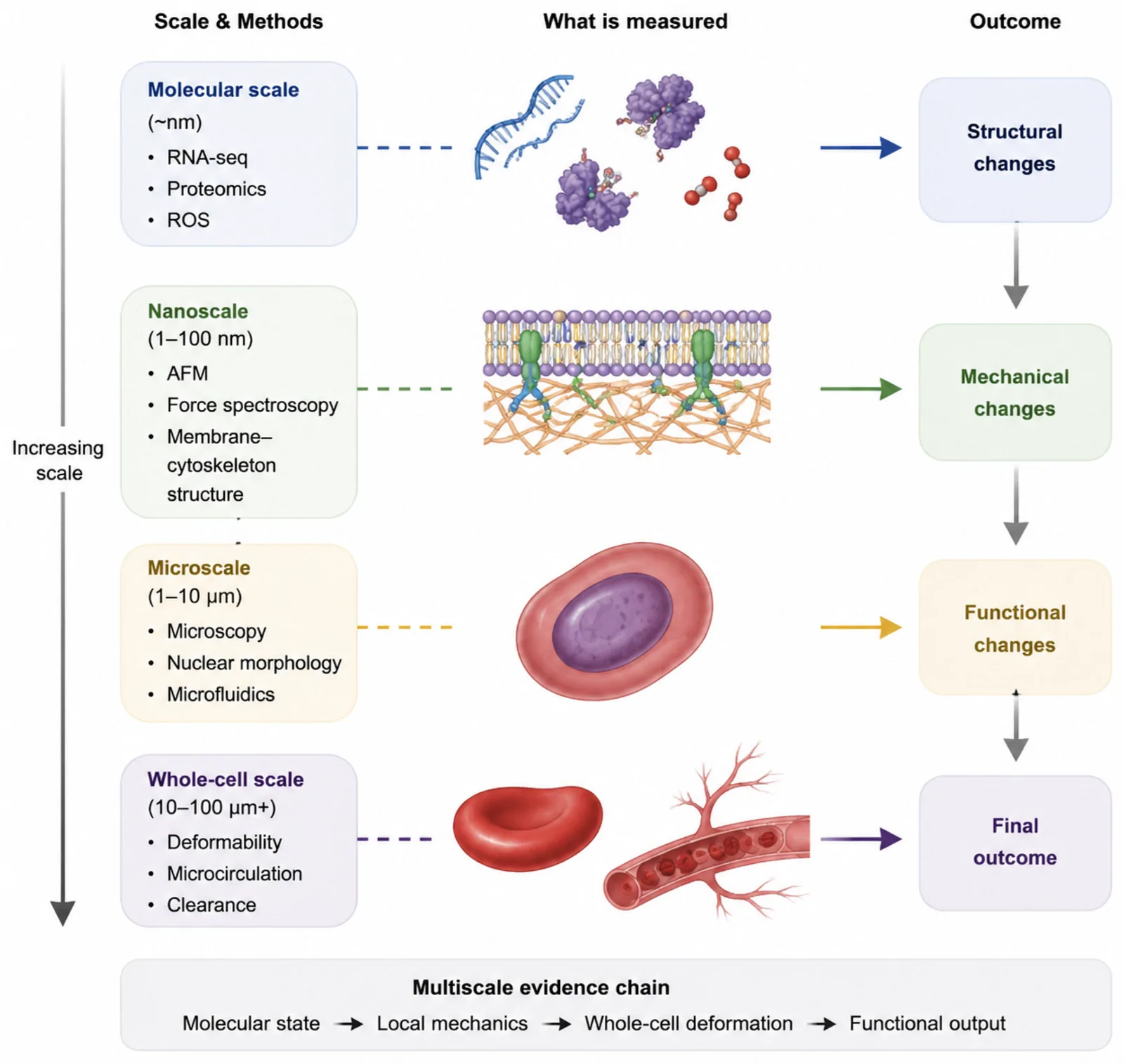
Multiscale experimental strategy for linking structure, mechanics, and function in nucleated erythrocytes.

The core of multi-scale research is not how many technical means are used, but whether the measurement results of different scales can build a continuous and mutually supporting evidence chain around the same biological problem. Local morphology and material properties, whole cell deformation, molecular state and functional output were captured by different technologies [90,91,92]. If sample sources, time points, and experimental conditions are not aligned across studies, even significant changes in all measured parameters may not be sufficient to demonstrate how structural alterations are translated into mechanical abnormalities and subsequently influence cellular function [93]. Liang et al. compared different measurement techniques for assessing erythrocyte deformability and pointed out that micropipette aspiration, optical tweezers, AFM, ektacytometry, and other methods differ in terms of stress mode, measurement scale, and output parameters [3]. Therefore, based on the basic principles of living cells, near-physiological liquid phase, homologous samples and time series, the structure/molecular state, local mechanics, whole cell deformation and functional output can be connected in series into the same evidence chain [93,94]. Such an integrated multiscale experimental strategy provides an approach for systematically clarifying relationships among erythrocyte structure, mechanics, and function, as summarized in Figure 4.

### 7.1. Characterization of Structure and Molecular State: Microscopic Imaging and Histology Provide Space and Mechanism Baseline

Bright-field and phase-contrast microscopy are suitable for rapidly measuring cell major and minor axes, cell area, nuclear-to-cell area ratio, and the proportion of abnormal morphologies, providing the geometric baseline for subsequent mechanical analyses [94,95]. Fluorescence and confocal microscopy can further visualize the distribution of molecular signals, including F-actin, membrane proteins, chromatin organization, mitochondrial structures, and ROS signals [96]. Scanning electron microscopy(SEM) and transmission electron microscopy(TEM) provide high-resolution structural evidence of membrane surfaces, organelles, and pathogen–cell interactions [97]. Majstorović et al. demonstrated the phagocytosis of particles by carp erythrocytes using confocal microscopy and TEM, indicating that high-resolution spatial evidence is particularly important for distinguishing true intracellular uptake from simple surface adhesion in infection studies [41]. Cell thickness, nuclear height, and nuclear position can be considered as geometric parameters when interpreting AFM indentation and microfluidic deformation data, to avoid misinterpreting thickness effects caused by elevation of the nuclear region as intrinsic differences in material stiffness [98].

Nucleated erythrocytes retain some transcriptional activity, so transcriptome, epigenome and proteome analysis can further reveal the molecular mechanisms behind their structural changes [20,64]. Methylation studies in chicken red blood cells and transcriptional evidence in avian and fish infection models suggest detectable nuclear regulatory activities in nucleated red blood cells, and the corresponding omics experiments can strictly control leukocyte contamination and cell purity [20,30,64]. Analyses directly related to mechanical issues can be prioritized around modules such as membrane skeleton, REDOX, ion transport, heat shock response, mitochondrial quality control, inflammation, and cell death [20,64,88,96]. Omics approaches are more suitable for generating candidate mechanisms and can be validated through structural and mechanical phenotyping as well as targeted interventions. Mechanical causality cannot be established from differential expression alone.

### 7.2. Local Mechanical Characterization: Atomic Force Microscopy (AFM) Mechanical Mapping and Its Model Boundary

AFM can obtain surface morphology and force–distance curve at the same time on the nanometer scale, which is a potential key technology to analyze the spatial mechanical heterogeneity of nucleated red blood cells [99]. Previous AFM studies on erythrocytes have established the feasibility of quantitatively linking nanoscale structural characteristics with mechanical properties. Girasole et al. demonstrated that AFM provides a quantitative approach for investigating native and pathological erythrocytes by correlating surface morphology and nanoscale organization with cellular functional changes [100]. More recently, Sergunova et al. summarized AFM-based characterization of red blood cells and highlighted its ability to evaluate cell morphology, membrane nanostructure, cytoskeletal organization, and mechanical stiffness, providing important methodological support for identifying erythrocyte mechanical heterogeneity [101]. These studies further support the application of AFM as a spatially resolved tool for understanding relationships between structure and mechanics.

AFM measurements of nucleated erythrocytes reveal pronounced spatial and methodological dependence in apparent stiffness. Baier et al. reported median apparent Young’s moduli of 2278 Pa above the nucleus and 461 Pa in the peripheral region of chicken erythrocytes at 25 °C, with corresponding values of 2384 and 183 Pa at 42 °C, demonstrating strong nucleus-related mechanical heterogeneity [51]. Guzniczak et al. reported 1.04 ± 0.47 kPa for nucleated human erythroid cells measured by AFM with a conical probe in Hanks’ balanced salt solution (HBSS) at 37 °C [50]. These values need not be treated as directly comparable intrinsic material constants because probe geometry, indentation depth, force range, medium, temperature, cell attachment, and sampling location can all affect the extracted modulus. Finite-thickness and substrate effects are particularly important in the thin erythrocyte periphery.

However, there is still a lack of the same systematic mechanical data of nucleated erythrocytes in poultry and fish. Therefore, the phenomenon that the mechanical properties of the nuclear region are higher than those of the cytoplasmic region is not regarded as a cross-species universal law. Compared with only reporting a single cell Young’s modulus, a more informative strategy is to construct a spatial registration mechanical map of the nuclear center, nuclear edge, cytoplasmic region and cell edge, and simultaneously record the cell thickness, nuclear height, indentation depth and loading conditions [99,102,103]. For nucleated red blood cells, near physiological liquid phase, low load and moderate limit can be given priority. High-speed AFM can be used in dynamic processes, but its stability on whole-cell nucleated red blood cells, the influence of cell fixation methods on the results and cross-laboratory repeatability still need special verification [99,104].

The interpretation of AFM data can match the heterogeneous geometry of nucleated red blood cells. The Hertz/Sneddon model usually assumes that the sample is homogeneous, isotropic, linear and elastic, and its thickness is much greater than the depth of penetration, but nucleated red blood cells with a raised central nucleus and thin cytoplasm edge do not fully meet these conditions [103,104]. When the pressure is too deep, the obtained value will mix the membrane tension, membrane skeleton, cytoplasm, nucleus and basal effect, so it is more appropriate to express it as apparent Young’s modulus and clearly fit the hypothesis [103,104,105]. Therefore, although AFM-derived mechanical parameters provide valuable information on relationships between erythrocyte structure and mechanics, their interpretation in nucleated erythrocytes can consider nuclear geometry and cellular heterogeneity.

It is recommended to report, at a minimum, the probe geometry and stiffness, loading rate, maximum load, medium and temperature, fixation method, test area, indentation depth, contact point detection algorithm, and fitting model. Measurements of the nucleus and cytoplasm regions can be performed within the same cell, and the ratio of indentation depth to local thickness can be reported to assess substrate effects. If the research goal is to quantify the independent contribution of the nucleus to the overall mechanical properties, the information of the nucleus height or three-dimensional morphology can be obtained simultaneously.

### 7.3. Overall Deformation and Circulation Function: Microfluidic, Deformation Measurement and Controlled Mechanical Manipulation

AFM mainly describes local indentation mechanics, whereas microfluidic and population-based deformability measurements are more directly related to the functional behavior of erythrocytes during circulation [3,62]. Kajitani et al. used narrow microchannels to establish the Transit Velocity Index for evaluating erythrocyte deformability [106]. Du et al. further quantified mechanical fatigue induced by repeated deformation using a biomimetic circulation platform [107]. Xu et al. showed that microscale manipulation of erythrocytes has expanded beyond simple transport toward deformation characterization, cell sorting and multiparametric single-cell analysis [62]. These approaches are particularly relevant to nucleated erythrocytes, in which nuclear size and orientation, cell geometry and maturation state introduce additional sources of mechanical heterogeneity.

Recent microfluidic studies provide functional evidence that nuclear retention is associated with impaired erythroid-cell deformability, although they do not isolate the mechanical contribution of the nucleus itself. Oshabaheebwa et al. used a biomimetic microcapillary network to analyze in vitro-derived erythroid cells and found that the slow-cell fraction decreased from 67.2 ± 6.4% in a heterogeneous population containing nucleated cells to 32.9 ± 0.96% after enrichment of enucleated cells, while mean transit velocity increased from 0.45 ± 0.05 to 0.79 ± 0.05 mm s^−1^ [108]. Guzniczak et al. similarly showed deformability-dependent hydrodynamic differences between nucleated and enucleated erythroid cells in a 170 × 30 μm spiral microchannel. Enucleated and nucleated cells occupied lateral positions of 36 ± 21 and 103 ± 32 μm from the outer wall, respectively, enabling an estimated 96% separation of nucleated cells from the enucleated population [109]. Together, these findings associate nuclear retention with reduced deformability and altered microchannel transit, while indicating that the nucleus cannot be considered the sole mechanical determinant.

Microchannel geometry can therefore be selected according to the specific mechanical question. Fixed-width channels are suitable for comparing transit performance, tapered channels can identify critical confinement dimensions, and bifurcated networks allow analysis of cell partitioning. Repeated contraction–expansion structures are particularly useful for reproducing recurrent mechanical loading and fatigue [62,107]. Complementary techniques provide different levels of mechanical information: optical tweezers and micropipette aspiration can apply controlled forces to quantify membrane stretching, recovery and adhesion, whereas ektacytometry provides population-level assessment of deformability [3]. The time-dependent oxidation–shear measurements reported by Larkin et al. further suggest that dynamic responses may provide more information about mechanical stress resistance than single endpoint measurements [54]. Microfluidic systems may therefore be integrated with controlled hypoxia, temperature or ROS exposure to compare deformation behavior under standardized flow conditions.

For nucleated erythrocytes, future microfluidic measurements can resolve both cellular and nuclear behavior during transit. High-speed imaging could quantify entry, maximum deformation, nuclear orientation, exit and post-passage recovery, while minimally perturbative nuclear labeling, where feasible, could help distinguish deformation of the cell boundary from internal nuclear constraints [110]. Channel dimensions can be reported relative to both cell and nuclear dimensions, together with transit time, deformation index, nuclear orientation, post-passage recovery, morphology, maturation state and relevant physiological conditions. Such reporting is necessary to distinguish genuine biological variation from differences introduced by experimental design.

Overall, functional mechanical assessment can preserve dynamic information and be interpreted together with local AFM measurements and structural or molecular parameters. No single mechanical parameter can be treated as a direct surrogate for microcirculatory function.

### 7.4. Multimodal and Time Series Integration: From Parallel Indicators to Causal Evidence Chain

The major value of multi-scale approaches lies in integrating spatial, mechanical, molecular, and functional information within the same biological context. Ideally, imaging, AFM-based mechanical mapping, and functional measurements should be combined across defined stages, including baseline, early stress, remodeling, recovery, or irreversible damage, to distinguish adaptation from injury and establish temporal relationships among stress, structural changes, mechanical responses, and functional outcomes [111,112]. Data analysis should follow this hierarchical framework by integrating structural, mechanical, biochemical, and functional variables while considering the nested nature of single-cell measurements [113,114]. Importantly, correlation alone cannot establish causality; mechanistic conclusions require temporal evidence and intervention-based validation linking upstream molecular or structural changes with downstream mechanical and functional phenotypes [115]. Therefore, future studies should prioritize integrated workflows combining spatial registration, dynamic monitoring, hierarchical analysis, and causal validation. The major technologies and their recommended combinations are summarized in Table 4.

## 8. Key Scientific Issues, Evidence Gaps and Future Directions

At present, the main limitation in this field is no longer the lack of function, but the causal chain between structure, mechanics and function is still incomplete. There is no unified answer to how much mechanical constraint the nucleus contributes, how stress and immune activation may affect erythrocyte function in the circulation, whether the results obtained by different species and technical platforms can be compared, and which indicators have stable transformation value. Future research can focus on four directions: causal verification, cross-scale and cross-species standardization, falsifiable hypothesis and evidence classification, and transformation and connection.

### 8.1. Core Mechanism Gap: From “Nucleated” Phenotype to Structure-Mechanics-Function Causality

The most basic and unsolved problem is the actual contribution of the nucleus and its chromatin to the overall mechanical behavior of mature nucleated red blood cells [119,120,121]. The key is to simultaneously quantify nuclear area, height, orientation, and chromatin state, together with nuclear–cytoplasmic mechanics and whole-cell transit and recovery behavior, while controlling for cell size and external conditions [122]. In addition to comparing the apparent modulus of nuclear region and cytoplasmic region, it can also be tested whether the nuclear scale can independently explain transit time or recovery kinetics after controlling the total size, thickness and osmotic state of cells [123]. If the nuclear scale effect disappears after these covariates are corrected, the original explanation of nuclear limitation can be revised to global geometric effect, instead of continuing to strengthen the causal effect of the nuclear itself. Human mature red blood cells can only provide mechanical reference after enucleation, and causal judgment still needs intraspecific homologous measurements or interventions on nucleated red blood cells.

The second core gap lies in the interaction between stress and circulatory function. Combined stress and immune activation can change the state of membrane–skeleton, cytoplasm and nucleus at the same time, but it is still unclear when these active responses cross the adaptation threshold and turn into decreased deformability, increased adhesion or impaired microcirculatory function. Future research can focus on the stages of adaptation, sub-injury, clearance or death, so as to avoid confusing the early protective response with the injury outcome after continuous exposure.

### 8.2. Comparability of Evidence: Establish a Cross-Scale, Cross-Laboratory and Cross-Species Standard Model

The main reason why the existing mechanical evidence is difficult to integrate is the lack of unified standards for measurement conditions and statistical levels. Different studies use fixed or dried samples, different probes, loading rate, indentation depth and fitting model, and the obtained Young’s modulus cannot be directly compared laterally. This problem is particularly prominent for nucleated red blood cells with a raised central nucleus and a thin cytoplasm edge. Cross-laboratory comparisons can prioritize relative indices obtained under standardized conditions, such as the mechanical ratio between the nuclear and cytoplasmic regions, standardized deformation indices, changes before and after stress, and recovery time. Experimental parameters, including temperature, osmotic pressure, fixation method, probe and loading conditions, indentation depth-to-local thickness ratio, and fitting assumptions, can be reported in full [116,117,118]. The inclusion criteria for cells, such as maturity, abnormal morphology, nuclear location and cell thickness range, can also be specified in advance to avoid systematic deviation caused by differences in cell screening in different laboratories. Statistical analysis should follow the hierarchical structure of animals, samples, cells and intracellular regions to model, strictly distinguish biological duplication from technical duplication, and report the effect quantity and its confidence interval at the same time, instead of relying solely on *p* value to judge statistical significance.

Cross-species comparisons can also account for confounding factors related to phylogeny, ecology, and cellular scale. The analysis by Udroiu across 513 vertebrate species showed that red blood cell volume is associated with metabolic rate, hemoglobin oxygen affinity, and genome size in nucleated groups. Therefore, cell and nuclear dimensions, temperature, and phylogeny can be considered as covariates in cross-species comparisons [9]. A more reliable approach is to conduct comparisons first within species, followed by comparisons within taxonomic groups and across groups, while carefully reporting sampling and measurement temperatures, particularly for ectothermic animals [124]. Only after demonstrating that the same biophysical parameter has comparable interpretations across species can overall differences between nucleated and enucleated erythrocytes be discussed.

### 8.3. Transformation Prospect: From the Analysis of Human Erythroid Differentiation Mechanism to the Application of Blood Transfusion Medicine and Animal Health

The transformation value of nucleated red blood cells research is first reflected in the comparative reference to the differentiation and maturation process of human erythroid cells. Human erythroid precursor cells undergo nuclear condensation, cytoskeletal remodeling and nuclear extrusion before enucleation. Studies by Zhang et al. on PIM1 and Xu et al. on AURKA further demonstrate that nuclear-skeleton and vesicle dynamics are critical regulatory nodes for terminal erythroid enucleation [12,42]. Therefore, non-mammalian mature nucleated red blood cells, enucleated human erythroid precursors and enucleated human mature red blood cells can be regarded as three complementary states [125,126]. These three states can only be used to compare mechanical characteristics and the process of enucleation, rather than being regarded as directly causally equivalent across species. In the context of human erythroid differentiation and maturation, the value of nucleated red blood cells does not lie in modeling disease itself, but in providing a stable terminal cellular state that retains the nucleus while still performing circulatory functions. This complements the short-term enucleation stage represented by erythroid precursor models in vitro.

The second transformation direction involves the feasibility of mechanical phenotype as a state index. Membrane skeleton and metabolic damage can occur in human mature red blood cells under storage, inflammation and oxidation conditions, and microfluidic has been used to evaluate the quality of stored red blood cells [86]. The in vivo study of Islamzada et al. further suggests that low-deformability red blood cells which obviously deviate from the normal distribution can be selectively eliminated, indicating that the mechanical phenotype has potential information of circulating survival and elimination [44]. In birds and fish, heat stress and bacterial or viral infections have also demonstrated that red blood cells can exhibit active responses. If future studies demonstrate that certain deformability, recovery kinetics, adhesion, or nanomorphological indices exhibit stable temporal relationships during disease progression or stress responses, these parameters may serve as auxiliary functional phenotypes linking cellular injury with circulatory function [127,128]. Its application value is not to replace the existing immune or pathogenic detection, but to provide an integrated evaluation of cell function and circulation cost. Generally speaking, the key challenge for the next stage of this field is not to further expand the description of phenomena, but to develop nucleated red blood cells into a quantitative model that connects nuclear mechanics, erythroid biology, and comparative blood circulation physiology. This goal requires standardized, falsifiable, and causally oriented research designs.

## 9. Conclusions

Nucleated erythrocytes retain the nucleus, chromatin, and in several vertebrate groups functional organelles and transcriptional capacity, providing a structural basis for active regulation that is absent from mature human erythrocytes, which are therefore best used as an enucleated mechanical reference rather than a replacement model. This perspective further emphasizes that nucleated erythrocytes can be considered mature multifunctional circulating cells rather than evolutionary incomplete counterparts of enucleated mammalian erythrocytes. Available mechanical evidence supports a composite, context-dependent model rather than the assumption that nuclear retention universally increases whole-cell stiffness. The nuclear region can show marked local mechanical heterogeneity, while whole-cell stiffness depends on maturation state, cell/nuclear geometry, temperature, and measurement conditions. Avian and fish erythrocytes can mount metabolic, oxidative, immune, and programmed cell-death responses, but these functions are species- and model-dependent. Direct evidence linking these responses to changes in deformability, rheology, and microcirculatory performance remains insufficient. The principal evidence gap is the lack of causal integration across structural, mechanical, and functional scales. Future studies can measure structural or molecular remodeling, local and whole-cell mechanics, and functional circulatory outcomes longitudinally in the same system under standardized, near-physiological conditions. Establishing a reproducible “structural change–mechanical phenotype–functional outcome” chain would convert the current comparative approach into a testable mechanistic model.

## Figures and Tables

**Table 1:** Comparison of structural baseline between human mature red blood cells and main nucleated red blood cell models.

Comparative Dimension	Human Mature RBC	Avian RBC	Fish RBC	Amphibian/Reptilian RBC
Nucleus/organelles	Enucleated, basically without organelles [1,3]	Nucleated, with partial retention of organelles including mitochondria [17,40]	Nucleated with retained organelles [13,19]	Nucleated, large nuclear volume, and significant species differences [32]
Typical form	Double concave disk [3]	Elliptical/ovoid morphology with central nuclear elevation [28]	Predominantly elliptical/ovoid [13]	Predominantly elliptical with considerable size variation [29,32]
Key scale parameters	Diameter, thickness, surface area/volume ratio [3]	Long and short axes, nuclear area, and nucleus-to-cell area ratio [28]	Long and short axes, nuclear area, and cell area [13]	Nuclear-to-cell size scaling relationship [29,32]
Foundation of membrane–skeleton research	Best-supported high-resolution structural evidence [10,34]	Limited direct high-resolution evidence [17]	Membrane/surface structure evidence with limited systematic studies [13]	Limited direct structural and mechanical evidence [29,32]
Structural layer reference meaning	Simplified structural benchmark after core removal [1,10]	Typical model of nucleus–chromatin–organelle coexistence [17]	Nucleated and functionally active circulating cell model [13,19]	Key model for nuclear size and morphology comparison [32]

**Table 2:** Representative experimental evidence linking stress exposure, mechanical responses, and biological outcomes in chicken, fish, and human erythrocytes.

Species and Maturation Stage	Exposure Type and Duration	Measurement Method	Specific Results (Units)	Variability and Sample Size	Biological/Mechanical Outcome	Reference
*Emberiza bruniceps*; adult males (nMig/pMig/Mig)	Photoperiod-induced migratory stress (14–28 d)	Flow cytometry; mitochondrial assays; RT-qPCR	ROS MFI decreased (1037→756→834); apoptosis reduced (6.86%→2.26%); mitochondrial remodeling detected	*n* = 18 (6/group); mean ± SEM	Improved erythrocyte resilience through mitochondrial adaptation and oxidative stress regulation	[39]
*C. carpio*; adult nucleated erythrocytes	Bacterial PAMP stimulation (LPS/PGN; 24 h, in vitro)	Flow cytometry; microscopy; qPCR	Enhanced phagocytic activity and cytokine transcription (relative mRNA expression, fold-change)	*n* = 6 fish; independent biological replicates	Immune activation of nucleated RBCs; functional remodeling under infection stress	[41]
*H. sapiens*; mature RBCs	Hypoxic stress (95% N2/5% CO_2_; 120 min, in vitro)	Ektacytometry; Western blot; thiol oxidation assay	Reduced erythrocyte deformability index; increased band 3 phosphorylation and protein thiol oxidation (relative intensity)	*n* = 3 donors; independent experiments	Membrane protein oxidation disrupted RBC mechanical resilience	[65]
*G. gallus*; juvenile males (broiler lines and jungle fowl)	Acute heat stress (45 °C, 2 h)	qPCR; liquid crystal deformation assay	RBC deformation varied among lines; aspect ratio of strained cells ~1.0–3.0; HS modulated HSP and cytokine genes	*n* = 4/line; mean ± SEM	Heat-induced stress altered RBC mechanics and inflammatory phenotype	[66]
*S. salar*; adult erythrocytes	ISAV infection (viral stress; 24 h, in vitro)	Lectin-based glycan profiling; microscopy; flow cytometry	Increased lectin-binding fluorescence intensity (MFI), indicating enhanced terminal sialic acid exposure	*n* = 3 fish; infected and control groups	Membrane surface remodeling and altered erythrocyte–pathogen interactions	[67]
*C. fuscus*; adult erythrocytes	Bacterial infection (*A. hydrophila*; 4–8 h, in vitro)	RNA-seq; qPCR; cell death assays	Upregulated PCD-related genes (relative expression, fold-change) and activation of apoptosis/pyroptosis/necroptosis pathways	*n* = 3 fish/group; biological replicates	Programmed cell death activation and erythrocyte functional disruption	[68]

**Table 3:** Representative evidence from recent studies on stress responses and functions of nucleated erythrocytes.

Author/Year	Model/Stimulus	Main Research Methods	Main Findings	Relationship
Greene et al., 2022 [66]	Chicken RBCs, 45 °C heat stress	qPCR, liquid crystal mechanical strain	HSP/inflammatory gene and mechanical phenotype changes with strain and heat stress	Providing evidence for a stress–transcription–mechanical phenotype association
Bhardwaj et al., 2023 [39]	Migratory bird RBCs, metabolic state	Flow cytometry, mitochondrial membrane potential, qPCR	Coordinated changes in ROS, mitochondria, and antioxidant/lipid metabolism genes	Suggesting a possible metabolic–oxidative regulation pathway potentially relevant to cellular adaptation
Majstorović et al., 2024 [41]	Carp RBCs, *Aeromonas hydrophila*	qPCR, antibody, TEM/confocal	TNF-α/IFN-γ secretion, ROS, phagocytosis, and TLR upregulation	Suggesting possible links between immune activation and cellular functional responses
Tao et al., 2024 [68]	*Clarias fuscus* RBCs, *Aeromonas hydrophila*	Death/ oxidative markers	Multiple programmed cell death pathways and ferroptosis-related changes	Linking oxidative stress-associated damage with programmed cell death outcomes
Solarte-Murillo et al., 2025 [19]	Salmonid RBCs, PRV-1/PRV-3	Infection, qPCR, apoptosis assay	Host-specific differences in antiviral transcription and apoptosis	Demonstrating species-specific immune and cell-fate responses among nucleated RBCs
Liu et al., 2025 [18]	Chicken RBCs, APEC	IIF/SEM, qRT-PCR	Pathogen adhesion accompanied by temporal changes in complement-related genes	Directly linking pathogen adhesion to complement transcriptional responses

**Table 4:** Key techniques, core outputs, and recommended combinations for studying relationships among structure, mechanics and function in nucleated erythrocytes.

Techniques	Reference	Core Outputs	Advantages	Main Limitations	Recommended Combinations
AFM	[3,16,116,117,118]	Roughness, adhesion force, apparent Young’s modulus	Simultaneous measurement of nanotopography and local mechanics	Significant substrate/model/ fixation effects	Confocal microscopy, microfluidics
Microfluidics	[3,62]	Transit time, critical pressure, deformation and recovery	Close to microcirculation, single-cell high throughput	Difficulty in resolving local mechanical sources	AFM, high-speed imaging
Ektacytometry	[3]	Population elongation index and dynamic curves	High throughput, suitable for shear stress	Difficulty in resolving nuclear region heterogeneity	Flow cytometry, morphological imaging
Optical tweezers/ Micropipette aspiration	[3]	Membrane stretching, recovery, and adhesion	Precise force, dynamic manipulation	Low throughput	Microscopic imaging, modeling
Confocal/TEM	[41]	Spatial localization of cytoskeleton, nucleus, mitochondria, and pathogens	Direct structural evidence	Potential perturbation by fixation and labeling	AFM, omics
Transcriptomics/ Epigenomics	[20,39,64]	Stress pathways and regulatory networks	Unique mechanistic advantages of nucleated RBCs	Cell purity/causality issues	Mechanical phenotype association, intervention validation

## Data Availability

No new data were created or analyzed in this study. Data sharing is not applicable.

## Abbreviations

The following abbreviations are used in this manuscript:

AFM	Atomic force microscopy
ROS	Reactive oxygen species
RBC	Red blood cell
ATP	Adenosine triphosphate
BLVRB	Biliverdin reductase B
NADPH	Nicotinamide adenine dinucleotide phosphate
2,3-BPG	2,3-bisphosphoglycerate
HSP	Heat shock protein
TLR	Toll-like receptor
qPCR	Quantitative polymerase chain reaction
NLRP3	NLR family pyrin domain-containing protein 3
β-Cys93	β-globin Cys93
MMP	Mitochondrial membrane potential
PPP	Pentose phosphate pathway
NMR	Nuclear magnetic resonance
SEM	Scanning electron microscopy
TEM	Transmission electron microscopy
SICM	Scanning ion conductance microscopy
HBSS	Hanks’ balanced salt solution
